# Sectional Anatomy Quiz - III

**DOI:** 10.22038/AOJNMB.2018.33101.1228

**Published:** 2019

**Authors:** Rashid Hashmi

**Affiliations:** Rural Clinical School, University of New South Wales (UNSW), Wagga Wagga, NSW, Australia

**Keywords:** Anatomy, Computed tomography, Thorax

## Abstract

This series comprises of a quiz pertaining to the identification of salient and important anatomical structures and landmarks expected to be seen at a given level on the computed tomography (CT) image. The representative image is followed by a series of images showing examples of different commonly encountered pathological entities that can be seen at this level in a routine clinical practice. Readers are encouraged to identify highlighted anatomical structures and landmarks in all the images and appreciate how a given abnormality can alter the appearance of normal structures. It is expected that this series will help nuclear physicians in interpretation of the CT component of the single photon emission computed tomography (SPECT) and positron emission tomography (PET) studies.

***Answer***

The image is through the superior mediastinum approximately at the level of the 3rd thoracic vertebra (T3). 

A: Right brachiocephalic vein

B: Brachiocephalic (innominate) artery

C: Left common carotid artery

D: Left brachiocephalic vein

E: Left subclavian artery

F: Esophagus

G: Trachea 

Right 1^st^ to 4^th^ ribs are marked by the respective numbers

**Points to Remember**

- Superior mediastinum refers to the part of the mediastinum which is located cranial to a horizontal plane drawn across the lower portion of body of the 4^th^ thoracic vertebra posteriorly to the manubriosternal joint (sternal angle) anteriorly. Its superior limit is defined by the superior thoracic aperture (also called thoracic inlet or outlet) which is defined by a plane running across the 1^st^ thoracic vertebra posteriorly and superior border of the manubrium anteriorly.
- Main contents of superior mediastinum include aortic arch and its branches (brachiocephalic, left common carotid and left subclavian arteries), right and left brachiocephalic veins, upper portion of superior vena cava, trachea, esophagus, thymus, thoracic duct, lymph nodes, vagus nerve, phrenic nerve and left recurrent laryngeal nerve.
- On an axial CT images through the superior mediastinum at and cranial to the level of 3^rd^ thoracic vertebra, five vascular structures (i.e. brachiocephalic artery, left common carotid artery, left subclavian artery, right brachiocephalic vein, and left brachiocephalic vein) are clearly identified anterior to the trachea.
- Brachiocephalic veins lie anterior and lateral to the arteries. Left brachiocephalic courses across the midline anterior to the rachea to join the right brachiocephalic vein to form the superior vena cava.
- Brachiocephalic (innominate) artery is the first branch of the aortic arch. It arises in the midline and then courses upwards toward the right crossing the trachea and finally dividing into the right common carotid and right subclavian arteries posterior to the right sternoclavicular joint.
- Left common carotid artery is the second branch to arise from the aortic arch. It courses upward through the superior mediastinum to the level of left sternoclaviuclar joint before entering the neck.
- Left subclavian artery, last branch of the aortic arch, courses on the left of the trachea to the level of the left sternoclavicular joint before exiting the thorax via root of the neck.
- Esophagus is readily identified posterior to the trachea due to presence of air. However, when collapsed, it can appear as a soft tissue density structure of varying appearance.

**Figure 1:**
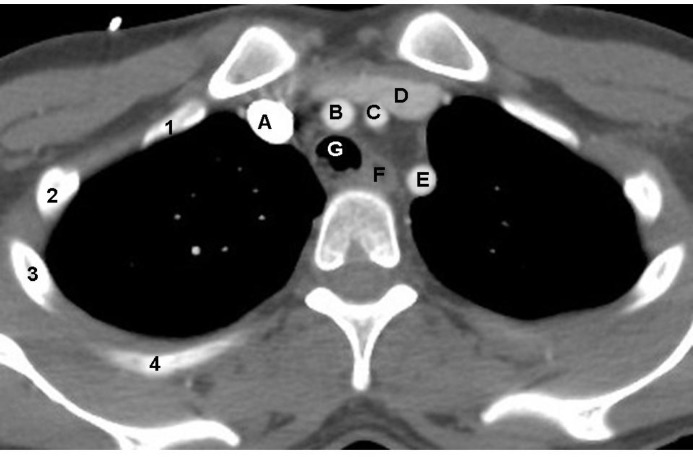
On contrast enhanced axial CT image of the chest of a 41 years old man shown above, identify the labelled normal anatomical structures labelled A to G.

**Figure 2:**
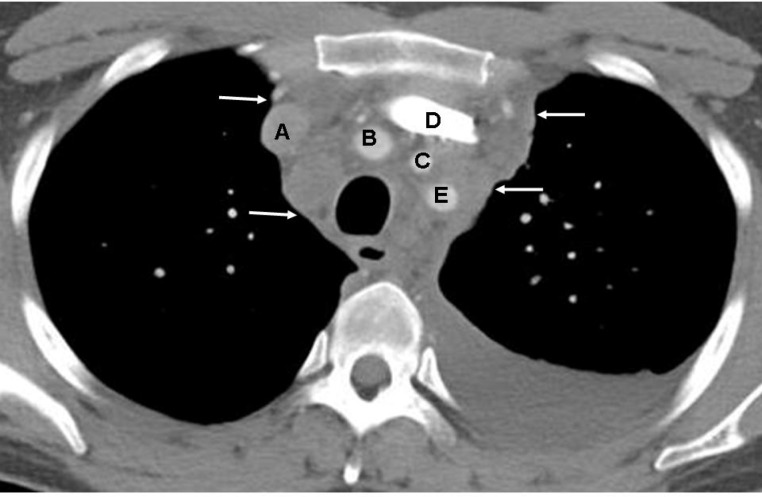
Contrast enhanced CT of the chest of a 25 years old male with Hodgkin lymphoma shows a large confluent mass (arrows) in the anterior mediastinum encasing, but not compressing, the right and left brachiocephalic veins (A and D respectively), brachiocephalic artery (B), left common carotid artery (C) and left subclavian artery (E). Prominent opacification of the left brachiocephalic vein is due to injection of the radiographic contrast in the left arm. Left sided pleural effusion is also seen

**Figure 3:**
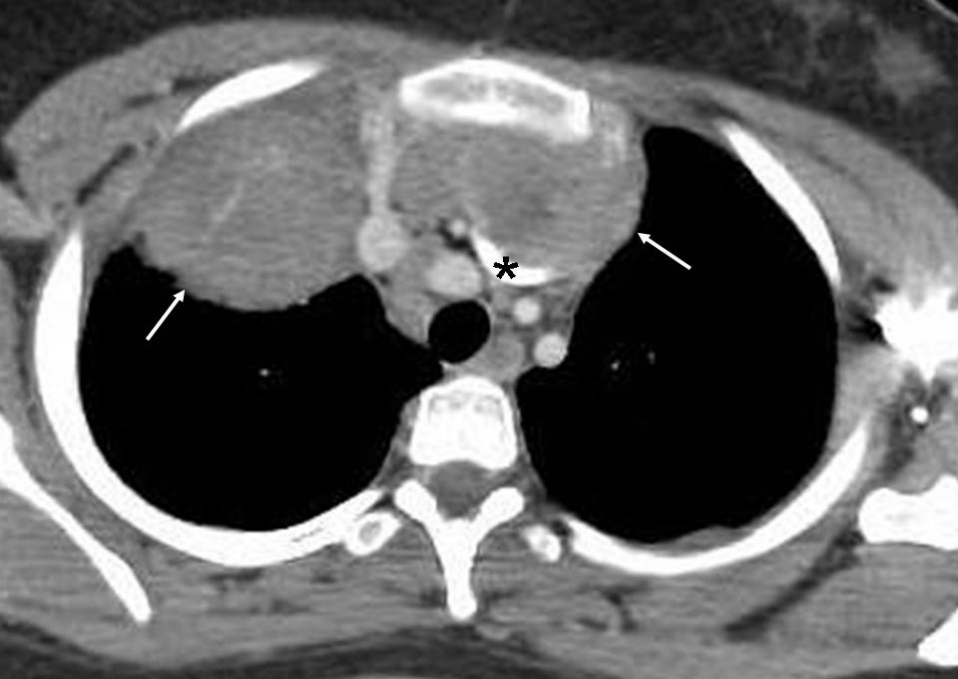
Contrast enhanced CT of the chest obtained in a patient with Hodgkin lymphoma shows a confluent mass (arrows) in the mediastinum anteriorly. The mass indents the superior aspect of the left brachiocephalic vein (asterisk). Small amount of pleural effusion is seen on the left side. It is to be noted that a mass originating in the mediastinum forms an obtuse angle with the adjacent lung, often has a smooth contour and can extend across the midline involving both hemithoraces. On the other hand, an intra-pulmonary mass forms an acute angle with the adjacent lung, does not extend across the midline, and may contain an air-bronchogram

**Figure 4:**
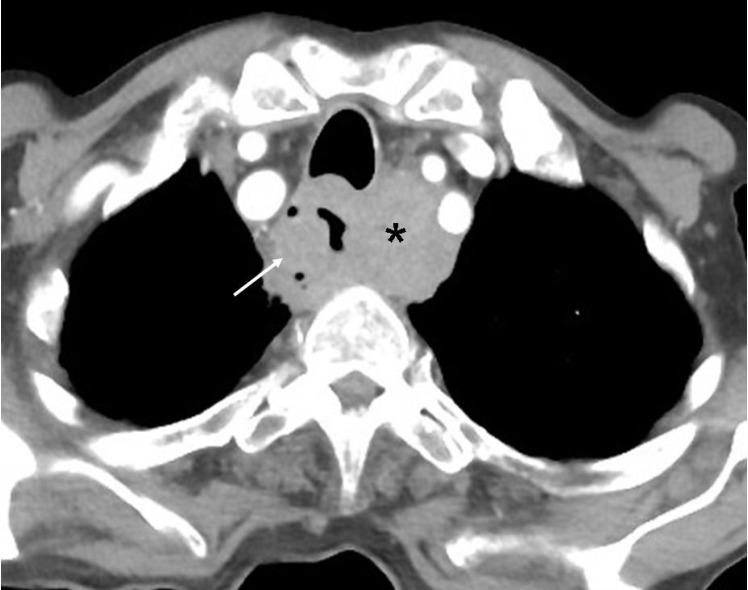
Contrast enhanced CT of the chest of a 58 years old man with carcinoma esophagus shows a large exophytic mass (arrow) in the esophagus narrowing its lumen and a large lymph nodal metastasis (asterisk) in the left paraesophageal region. Small lucencies within the mass suggest necrotic changes. Note anterior displacement of the trachea by the mass.

**Figure 5:**
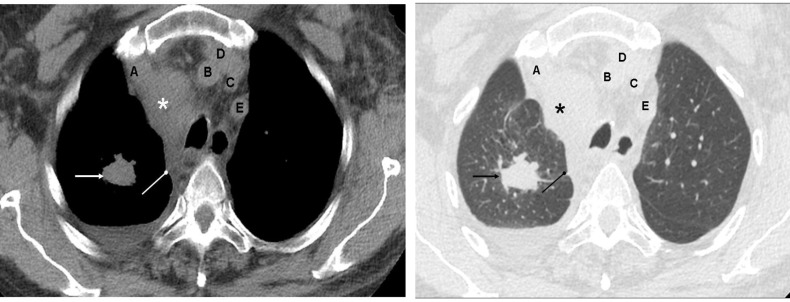
Mediastinal (first image) and lung (second image) windows of a non-contrast CT of the chest of a 55 years old smoker with bronchogenic carcinoma show the primary tumor in the right upper lobe (arrow), enlarged right upper paratracheal (asterik) and retrotracheal (oval arrow) lymph nodes and right sided pleural effusion. Speculated margin of the tumor is better appreciated on the lung window. It is to be noted that pulmonary pathologies (e.g. carcinoma, pneumonia, bronchiectasis etc.) are best assessed on the lung window while extra-pulmonary pathologies (e.g. lymphadenopathy, mediastinal tumor, hematoma, aortic dissection, pulmonary embolism etc.) are best appreciated on the mediastinal window. Vessels are labelled as A to E using same nomenclature as in Figure 1

**Figure 6:**
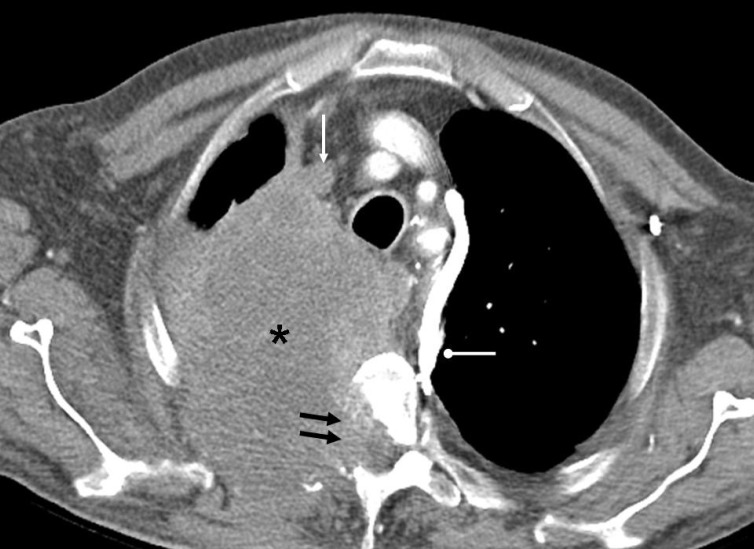
Contrast enhanced CT of the chest of a 64 years old male smoker with a large mass (asterisk) in the apical portion of the right lung shows absence of contrast in the right brachiocephalic vein (white arrow) suggesting tumor thrombus. Other vessels are well opacified. Mass destroys right side of the vertebral body and adjacent rib and extends into the spinal canal (double black arrows). Dilated hemi-azygous vein (oval arrow) is noted along the left side of the mediastinum suggesting collateral flow

**Figure 7:**
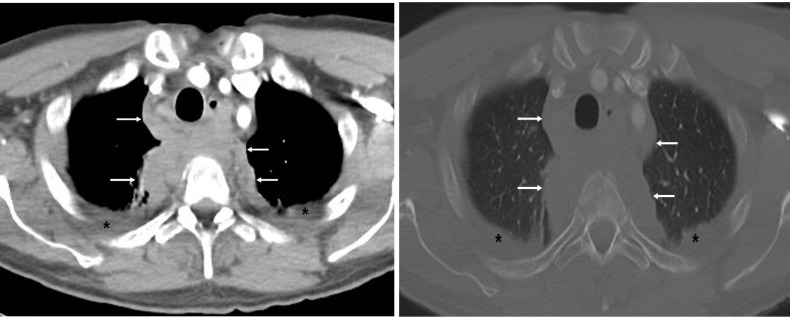
Mediastinal (first image) and bone (second image) windows of contrast enhanced chest CT of a 26 years old male with multiple thoracic vertebral fractures sustained after a high speed road traffic accident show a large para-vertebral high tissue density (arrows) suggesting mediastinal hematoma. Esophagus is displaced anteriorly to the left side of the trachea by the hematoma. Bilateral pleural effusion (asterisk) is also present. Vertebral fractures are not visible on these images

**Recommendations for Further Reading**

1. Ryan S, McNicholas M, Eustace SJ. Anatomy for diagnostic imaging e-book. 3rd ed. New York: Elsevier Health Sciences; 2010.
2. Olivetti L. Atlas of imaging anatomy. Berlin: Springer; 2015.
3. Currie S, Kennish S, Flood K. Essential radiological anatomy for the MRCS. Cambridge: Cambridge University Press; 2009.
4. Moeller TB, Reif E. Pocket atlas of sectional anatomy. CT and MRI. Thieme Stuttgart: Thorax, Abdomen, and Pelvis; 2001.

